# The Effect of Body Mass Index on Peri-operative Parameters of Total Laparoscopic Hysterectomy: An Institutional Experience

**DOI:** 10.7759/cureus.15558

**Published:** 2021-06-09

**Authors:** Kavita Khoiwal, Nirali Kapoor, Amrita Gaurav, Rupendra K, Kranti Kumar Reddy, Jaya Chaturvedi

**Affiliations:** 1 Obstetrics and Gynaecology, All India Institute of Medical Sciences, Rishikesh, IND; 2 Preventive Medicine, All India Institute of Medical Sciences, Rishikesh, IND

**Keywords:** body mass index, obese, overweight, normal weight, total laparoscopic hysterectomy

## Abstract

Objectives

Worldwide, there is an increase in the obese population and laparoscopic surgery is now becoming one of the preferred modes of surgery. Therefore, it is important to examine its feasibility and safety in overweight and obese women. The study was aimed to evaluate the effects of body mass index (BMI) on intraoperative and postoperative parameters in patients undergoing total laparoscopic hysterectomy (TLH).

Materials and methods

A retrospective data analysis was conducted over a period of two years among women who underwent TLH. Data were grouped as per their BMI into Normal, Overweight, and Obese groups. Baseline demographic and clinical characteristics, intraoperative outcomes including operative time, estimated blood loss, hemoglobin difference, the need for blood transfusion, conversion to laparotomy, uterine weight, intraoperative and postoperative complications, postoperative pain, duration of hospital stay, and readmission were noted.

Results

Baseline demographic characteristics were similar in all three groups. Operative time was comparable among the groups. However, a weak positive correlation was found between operative time and total BMI value, which was statistically significant (p = 0.039). For every 1 kg/m2 increase in BMI, operative time increased by 2.35 minutes. Other intraoperative parameters were comparable among all the groups except hemoglobin difference, which was significantly higher in obese women. Postoperative parameters were similar among all groups.

Conclusion

Total laparoscopic hysterectomy is a safe, efficient, and feasible surgical approach in higher BMI groups. This approach provides advantages over open surgery of early recovery, short hospital stay, and less postoperative pain to obese women.

## Introduction

Obesity has been declared a global epidemic by the World Health Organization (WHO). The prevalence of obese and overweight people is rising worldwide. It is more common in developed countries than in developing countries. However, 20.6% of women 15 to 49 years and 18.9% of men 15 to 49 years in India were overweight or obese in 2015 to 2016 according to National Family Health Survey India 4 [1]. The prevalence of overweight and obese women was higher in urban areas (31.3%) than in rural areas (15%) and lower in people who were involved in agriculture or manual work [1].

The ideal mode of hysterectomy in obese women is vaginal, similar to normal-weight women, followed by laparoscopic hysterectomy if a vaginal approach is not feasible [2]. A few years ago, laparoscopy was relatively contraindicated in obese women, but with the evolution in surgical techniques and expertise of surgeons and anaesthetists, it is now being performed in obese women. Women undergoing abdominal hysterectomy are more prone to incisional complications, wound infections, and wound dehiscence, which can be obviated with the laparoscopic route [3]. Additionally, laparoscopic hysterectomy is associated with early postoperative recovery, less postoperative pain, and early ambulation, which further reduces the risk of deep venous thrombosis and pulmonary embolism known to occur in the postoperative period in obese women [4]. However, it may be associated with longer operative time [3]. Total operative time has been found to increase in all overweight and obese women despite the route of surgery [3]. The surgical challenges of laparoscopy in obese women include creation and maintenance of pneumoperitoneum and inadequate Trendelenburg position owing to associated cardiorespiratory compromise.

High body mass index (BMI) has been determined to be an independent risk factor for laparoscopic hysterectomy, affecting surgical outcome and increasing the risk of intraoperative and postoperative complications [5,6]. However, the results of articles studying the effect of BMI on surgical outcomes of laparoscopic hysterectomy are incoherent. Some authors found significantly longer operation time and more perioperative complications in obese women than in patients with normal weight [6-8], whereas others documented no significant difference among BMI groups [9-11]. In addition, a significant reduction in complications has also been described in obese women than in normal-weight women [12].

In India, there is an increase in the obese population and laparoscopic surgery is now becoming one of the preferred modes of surgery. Therefore, it is important to examine its feasibility and safety in overweight and obese women. On that account, we conducted a retrospective analysis to evaluate the effect of BMI on perioperative outcomes in women undergoing total laparoscopic hysterectomy at a teaching institute in India.

## Materials and methods

This was a retrospective study conducted at the Department of Obstetrics and Gynaecology, All India Institute of Medical Sciences, Rishikesh, from March 2018 to February 2020. Data of 155 patients who underwent total laparoscopic hysterectomy (TLH) were collected. Seven patients were excluded from the analysis as an additional procedure was performed (two pelvic lymphadenectomies, two transobturator tape placements, one sacrocolpopexy, one cholecystectomy, one scar endometriosis excision). Informed and written consent was obtained from all included patients before their surgery. The study has been reviewed and approved by the institutional ethics committee (AIIMS/IEC/20/444).

Baseline demographic and clinical characteristics and intraoperative and postoperative outcomes were noted. We collected information on the patient’s age, parity, BMI, indication of hysterectomy, history of cesarean section or any other surgery, comorbidities, operative time, estimated blood loss, hemoglobin difference (preoperative - postoperative hemoglobin), need for blood transfusion, conversion to another approach (laparotomy), uterine weight, intraoperative and postoperative complications, pain in postoperative period, duration of hospital stay, and readmission.

All hysterectomies were performed under general anaesthesia in lithotomy position with pneumoperitoneum (12-15 mmHg). One 10 mm trocar (umbilical) and three 5 mm side trocars were used. A uterine manipulator was used during hysterectomy for manipulation. Step-wise transection of round ligament, dissection of bladder peritoneum, coagulation and transection of infundibulopelvic or ovarian ligament, uterine artery, cardinal and uterosacral ligaments was done and hemostasis was achieved. After colpotomy, the uterus was retrieved vaginally and vault closure was done laparoscopically.

The operative time was measured from the time of skin incision to skin closure. Estimated blood loss was measured from suction contents and gauze counting and deduction of amount of fluid used for irrigation purposes. Postoperative pain was measured on day 1 of surgery by visual analog scale (VAS). For postoperative pain relief, all patients were given IV paracetamol every eight hours for 24 hours followed by oral paracetamol every eight hours for 48 hours and thereafter on demand, as per the institutional protocol.

Intraoperative and postoperative complications were noted from case records or telephonically and follow-up was taken at 10 days, six weeks, three months, and six months post-surgery. Clavien-Dindo classification was used to grade complications [13].

The data were then divided into three groups according to BMI as per the WHO classification [14]: normal-weight group (18.5 - 24.9 kg/m2), overweight group (25 - 29.9 kg/m2), and obese group (>30 kg/m2) and subjected to statistical analysis.

Statistical analysis

Data were coded and recorded in Microsoft Excel. SPSS version 23 (IBM Corp., Armonk, NY, USA) was used for data analysis. Descriptive statistics were elaborated in the form of means/standard deviations and medians/IQRs for continuous variables, and frequencies and percentages for categorical variables. Data were presented in a graphical manner wherever appropriate for data visualisation using histograms/box-and-whisker plots for continuous data. Group comparisons for continuously distributed data were made using independent sample t-test when comparing two groups. If data were found to be non-normally distributed, appropriate non-parametric tests in the form of Wilcoxon Test were used. Chi-squared test was used for group comparisons for categorical data. In case the expected frequency in the contingency tables was found to be <5 for >25% of the cells, Fisher’s exact test was used instead. Linear correlation between two continuous variables was explored using Pearson’s correlation (if the data were normally distributed) and Spearman’s correlation (for non-normally distributed data). Statistical significance was kept at p < 0.05.

## Results

**Figure 1:**
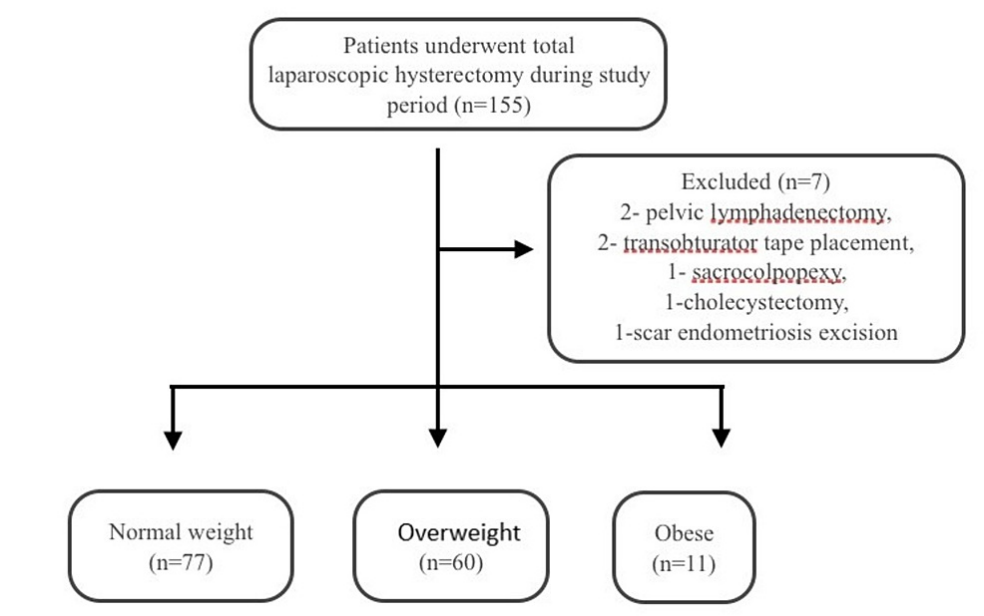
Flow chart of the study

A total of 148 women who underwent TLH were included in the analysis. Out of 148 women, 77 were classified into the normal-weight group, 60 women in the overweight group, and only 11 women in the obese group. No woman was found to be underweight (18.5 kg/m2). A flow chart of the study is shown in Figure 1.

The overall mean BMI in the study was 24.8 + 2.23 kg/m2.

Table 1 shows baseline demographic and clinical characteristics. The most common indication for TLH was abnormal uterine bleeding due to leiomyoma and adenomyosis. Mean age, parity, indication of surgery, and previous cesarean section were comparable in all three groups. Medical comorbidities were significantly more prevalent in higher BMI groups (p value = 0.019).

**Table 1:** Baseline demographic and clinical characteristics BMI - Body mass index; CS - Cesarean section ***Significant at p<0.05, 1: Kruskal Wallis Test, 2: Fisher's Exact Test, 3: Chi-Squared Test

Parameters	BMI Interpretation			p value
	Normal (n = 77)	Overweight (n = 60)	Obese (n = 11)	
Age (Years)	44.57 ±7.08	43.81 ±6.33	48.25 ±8.66	0.454^1^
Indication of surgery				0.086^2^
Leiomyoma	49 (63.6%)	46 (76.7%)	8 (72.8%)	
Adenomyosis	14 (18.2%)	3 (5%)	1 (9.1%)	
Adnexal Mass	7 (9.1%)	5 (8.3%)	0 (0.0%)	
Others	7 (9.1%)	6 (10%)	2 (18.1%)	
Parity	2.83 ±1.37	3.01 ±1.20	2.00 ±0.82	0.140^1^
Previous CS	8 (10.4%)	8 (11.9%)	3 (27.0%)	0.442^2^
Number of Previous CS	0.17 ±0.52	0.18 ±0.55	0.25 ±0.50	0.721^1^
Past history***				0.019^2^
No co-morbidities	52 (67.5%)	39 (65%)	5 (45.45%)	
Hypothyroidism	13 (16.9%)	4 (6.67%)	1 (9.09%)	
Hypertension	2 (2.6%)	7 (11.66%)	2 (18.1%)	
Diabetes Mellitus	1 (1.3%)	4 (6.67%)	1 (9.09%)	
Previous pelvic Surgery	1 (1.3%)	0 (0.0%)	1 (9.09%)	
Others	8(10.3%)	6 (10%)	1(9.09%)	

Intraoperative parameters

**Figure 2:**
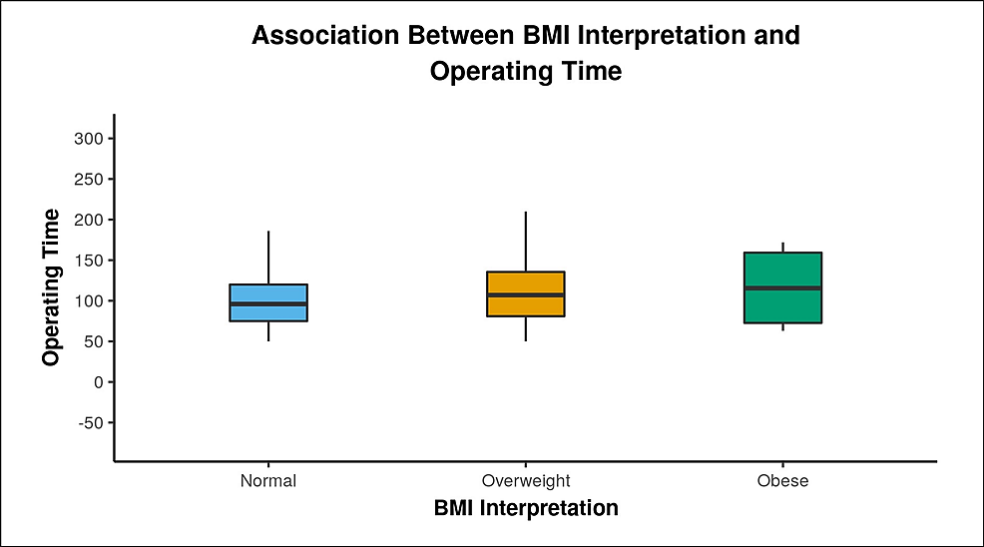
The Box-and-Whisker plot depicts the distribution of operative time in the three groups. The middle horizontal line represents the median operating time, the upper and lower bounds of the box represent the 75th and the 25th centile of operative time respectively, and the upper and lower extent of the whiskers represent the maximum and the minimum operative time in each of the groups.

**Figure 3:**
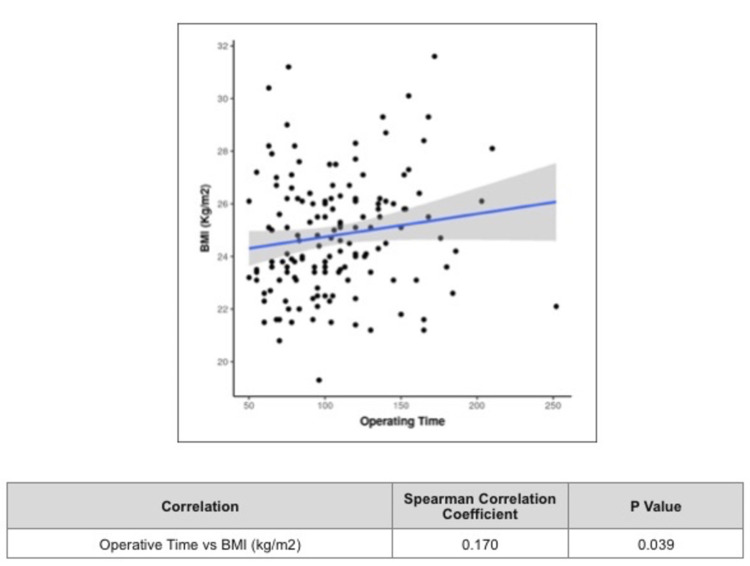
Scatterplot depicts the correlation between operative time and BMI (kg/m2). Individual points represent individual cases. The blue trend line represents the general trend of correlation between the two variables. The shaded grey area represents the 95% confidence interval of this trend line.

Table 2 shows intraoperative outcome variables. The mean operative time in the normal-weight group was 102.55 ± 36.91 minutes, the overweight group was 110.85 ± 35.12 minutes, and the obese group was 116.50 ± 54.97 minutes, not significantly different among individual BMI groups (p value = 0.216) as shown in Figure 2. However, there was a weak positive correlation between operative time and total BMI value (kg/m2), and this correlation was statistically significant (p = 0.039; Spearman correlation coefficient = 0.170). For every 1 minute increase in operative time, the BMI increased by 0.01 kg/m2. Conversely, for every 1 kg/m2 increase in BMI, the operative time increased by 2.35 minutes (Figure 3).

**Table 2:** Association between BMI and Intraoperative Parameters BMI - Body mass index ***Significant at p<0.05, 1: Kruskal Wallis Test, 2: Fisher's Exact Test, 3: Chi-Squared Test

Parameters	BMI Interpretation			p value
	Normal (n = 77)	Overweight (n = 60)	Obese (n = 11)	
Operative Time	102.55 ±36.91	110.85 ±35.12	116.50 ±54.97	0.216^1^
Blood Loss (ml)	134.83 ±74.85	150.37 ±75.54	135.00 ±51.96	0.207^1^
Change in Hemoglobin***	1.03 ±0.61	0.95 ±0.67	2.05 ±0.72	0.017^1^
Need of blood transfusion	9 (11.7%)	5 (8.3%)	0 (0.0%)	0.715^2^
Conversion to laparotomy	1 (1.3%)	1 (1.6%)	0 (0.0%)	1.000^2^
Uterine Weight (gms)	246.39 ±173.44	246.87 ±144.92	231.25 ±84.78	0.416^1^
Visceral injuries	0 (0.0%)	0 (0.0%)	0 (0.0%)	1.000^3^

Intraoperative blood loss was comparable in all the BMI groups (p value = 0.2), whereas mean hemoglobin difference was significantly higher in obese women (p value = 0.017) but comparable in normal-weight and overweight groups. Other operative variables like need for blood transfusion, conversion to laparotomy, and uterine weight were comparable in all three groups. Two patients, one in the normal-weight and one in the overweight group, required conversion to laparotomy due to uncontrolled intraoperative hemorrhage. No visceral injury was noted in any BMI group.

Postoperative parameters

Postoperative variables such as pain, duration of hospital stay, and complications were comparable in all the groups as shown in Table 3. All patients were advised ambulation from the evening of the day of surgery. However, we did not compare it between the groups. The overall complication rate in this study was 14.8% (5.4% in normal-weight, 9.4% in overweight, and 0% in obese women). Postoperative complications such as pyrexia, wound infection, urinary tract infection, and vaginal discharge were equally distributed in all the groups. One patient in the overweight group had a grade IIIa complication, readmitted on the 15th postoperative day for surgical intervention. No patients had any vaginal cuff complications (hematoma/dehiscence/abscess), wound-related complications, or dyspareunia at six weeks, three months, and six months follow-up visits.

**Table 3:** Association between BMI and postoperative parameters. BMI - Body mass index; VAS - Visual analog scale ***Significant at p<0.05, 1: Kruskal Wallis Test, 2: Fisher's Exact Test, 3: Chi-Squared Test

Parameters	BMI Interpretation			p value
	Normal (n = 77)	Overweight (n = 60)	Obese (n = 11)	
Postoperative complications				
Pyrexia	2 (I)	3 (I)	0	0.707^2^
Urinary complaints	3 (I)	5 (I)	1 (I)	0.579^2^
Urinary injury	0	1 (IIIa)	0	0.480^2^
Wound Infection	1 (I)	1 (I)	0	1.000^2^
Vaginal discharge	2 (I)	4 (I)	0	0.384^2^
Vault Hematoma/ Dehiscence/ abscess	0	0	0	1.000^3^
Postoperative pain on day 1 (VAS)	2.97 ±0.67	2.97 ±0.60	3.25 ±0.50	0.591^1^
Duration of stay (days)	2.79 ±0.68	2.99 ±0.62	3.00 ±0.82	0.124^1^
Readmission	0	1	0	0.480^2^

## Discussion

The present study documented no significant difference in operative time, blood loss, need for blood transfusion, or intraoperative and postoperative complications among different BMI groups. The mean hemoglobin difference was highest in obese women yet overall morbidity was not increased in these women. Furthermore, the complication rate was lowest in obese women. Though the operative time was comparable in all the groups, a weak positive correlation was found between operative time and total BMI value, with each 1 unit increase in BMI (kg/m2) operative time increased by 2.35 units.

In accordance with our study, Bhandari et al. reported no significant difference (p value = 0.069) in total operative time between non-obese (52.26 ± 20.75 minutes) and obese groups (56.86 ± 18.22 minutes) [9]. Similarly, O’Hanlan et al. reported comparable operative time in all BMI groups (153.7 + 48.7 minutes in normal-weight, 152.2 + 56.5 minutes in overweight and 164.9 + 42.32 minutes in obese; p value = 0.185) [10]. On the contrary, Ortiz et al. reported significantly higher operative time (p value = 0.001) in the obese group (145.0 ± 59.7 minutes) in comparison to normal-weight (108.4 ± 33.0 minutes) and overweight group (110.4 ± 44.0 minutes) [7]. They also reported significantly less intraoperative blood loss (p value = 0.002) and uterine size (p value = 0.006) in the normal BMI group than overweight and obese groups. Larger uterine size in higher BMI groups seems to be a confounding factor in their study [7]. However, no such difference in terms of intraoperative blood loss and uterine weight/size was noted in our study.

Bardens et al. [15], Siedhoff et al. [8], and Otake et al. [6] reported in their individual studies that increase in BMI is associated with significant increase in total operative time and intraoperative blood loss.

In a previous study by Sokol et al., obese women (BMI >30 kg/m2) had more than two-fold risk of unintended laparotomy [16], whereas no obese women, one overweight, and one normal-weight woman required conversion to laparotomy in our study. The postoperative pain, duration of hospital stay, and readmissions were comparable in all three groups in our study, which is consistent with the studies reported by Bhandari et al. [9], Siedhoff et al. [8], and Otake et al. [6].

Immediate postoperative complications like pyrexia, urinary complaints, and infections were more common in the overweight group in our study though the values were not statistically significant. Siedhoff et al. [8] and Otake et al. [6] also described a similar frequency of complications in all BMI groups. However, the severity of complications was significantly influenced by BMI (p value = 0.01) in a study by Siedhoff et al. [8]. Bhandari et al. reported a higher frequency of minor complications in the obese group (p value = 0.001) [9]. The overall postoperative complications that occurred in our study were in 14.8% of cases (5.4% in normal-weight, 9.4% in overweight, and 0% in obese women). Similarly, Bardens et al. documented maximum postoperative complications in the overweight group (13%) than in normal-weight (11%) and obese women (3%) (p value = 0.008) [15]. In the present study, no minor or major complications happened in obese women. Only one woman in the overweight group had grade IIIa complication, which required readmission and surgical intervention.

One should avoid surgery by more appropriate medical treatment options whenever possible, to buy some time for weight reduction before surgery. Additionally, benefits of laparoscopic surgery should not be ignored due to potential risks in obese women. Benefits versus risks should be outweighed in each patient individually.

Our study suggests that laparoscopic hysterectomy in obese women is as safe and efficient as in normal-weight women. Potential risks of increase in operative time, blood loss, and perioperative complications could be dealt with by skilled pelvic surgeons and experienced anaesthetists. Studies have reported a decrease in operative time with an increase in experience for laparoscopic surgeries [17].

Non-inclusion of time spent in operative theatre apart from surgical time (anesthesia induction time), fewer patients in the obese group, and the retrospective nature of the study were possible limitations of this study. A predetermined sample size, randomization, and longer follow-up would have produced more robust results. Additionally, a comparison of outcomes with other modes of hysterectomy (e.g. abdominal or vaginal) with BMI-matched women would have added more value.

## Conclusions

To conclude, total laparoscopic hysterectomy is a safe, efficient, and feasible surgical approach in higher BMI groups. This approach provides advantages of early recovery, short hospital stay, and less postoperative pain to obese women. Simultaneously, it obviates the risk associated with abdominal surgery, i.e., wound infection and wound dehiscence.
